# Supplementary material for: MoCHI: neural networks to fit interpretable models and quantify energies, energetic couplings, epistasis, and allostery from deep mutational scanning data
Source: Genome Biol. 2024 Dec 2;25:303. doi: 10.1186/s13059-024-03444-y (PMC11610129; doi:10.1186/s13059-024-03444-y)
Supplement: Supplementary file 1 — Additional file 1: Supplementary figures 1-3. Supplementary figures related to Fig. 4 and Fig. 5. [file 13059_2024_3444_MOESM1_ESM.pdf]

Additional file 1

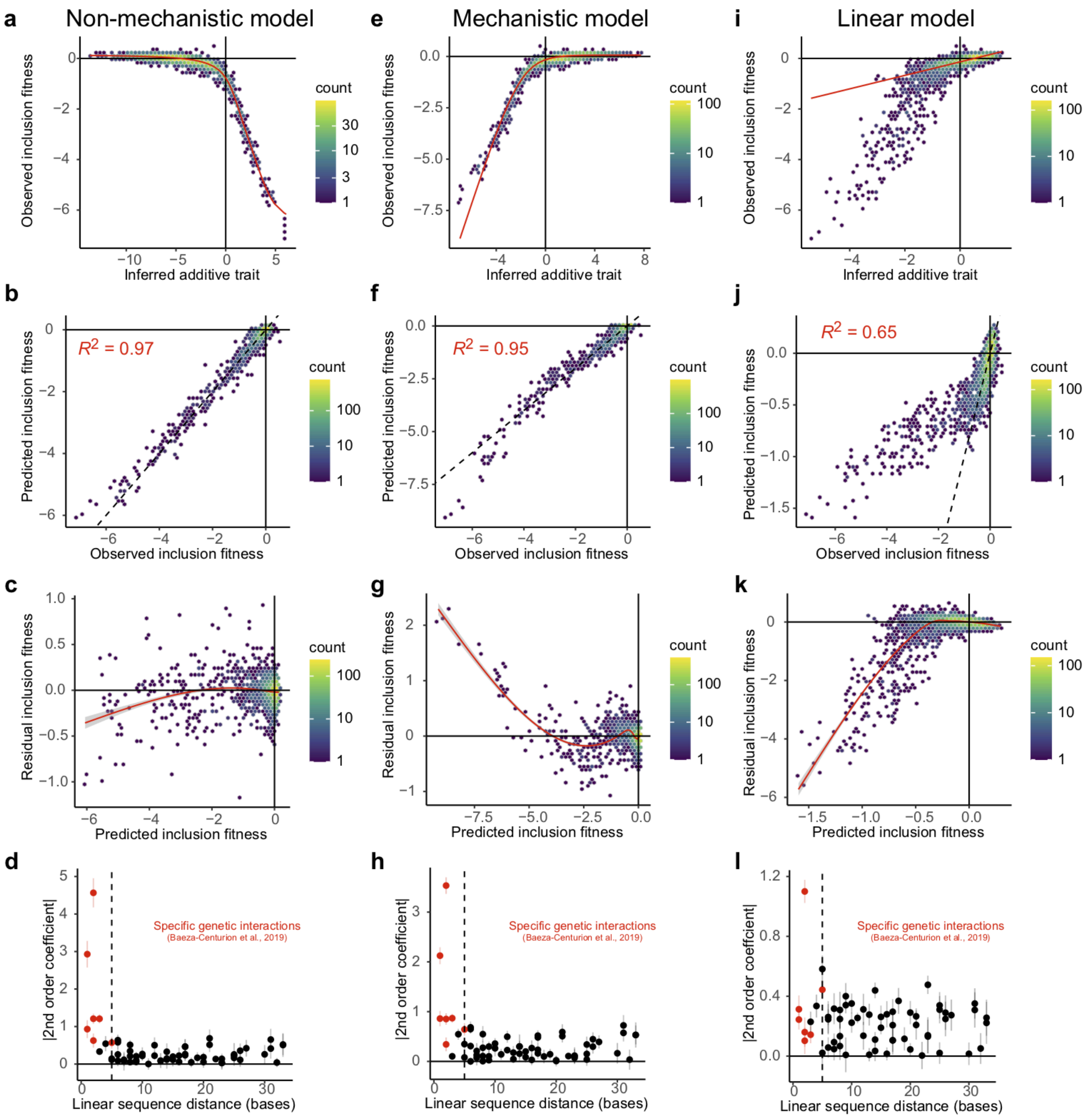

Fig S1. Related to Fig. 4, FAS exon 6. **a**, Inferred nonlinear relationship between observed inclusion fitness and the underlying additive trait (ϕ). **b**, Performance of 2nd order non-mechanistic MoCHI model predictions of inclusion fitness. **c**, Residual inclusion fitness (observed - predicted fitness) versus predicted inclusion fitness for the model in panel a. **d**, The magnitude of pairwise genetic interaction terms (2nd order coefficients) versus linear sequence distance separating the individual mutated positions in FAS exon 6. Red points indicate the top specific pairwise genetic interactions as described in[38] (see Fig. 4g). **e-h**, Similar to panels a-d but showing results for a mechanistic model of splicing competition[38] (see Methods). **i-l**, Similar to panels a-d but showing results for a linear model.

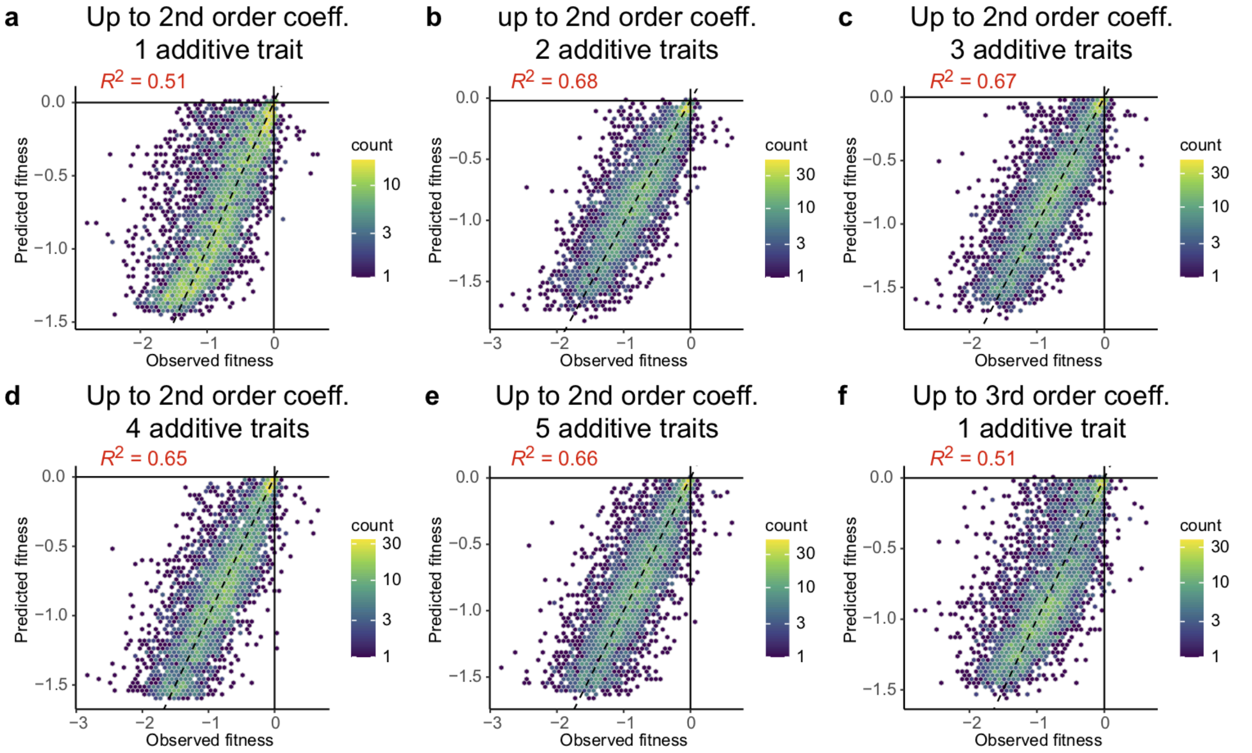

Fig S2. Related to Fig. 4, tRNA-Arg(CCU). **a**, Performance of 2nd order MoCHI model predictions of cellular fitness with a single inferred additive trait. **b-e**, Similar to panel a but models have 2-5 inferred additive traits (multi-dimensional epistasis). **f**, Similar to panel a but model has up to 3rd order epistatic coefficients.

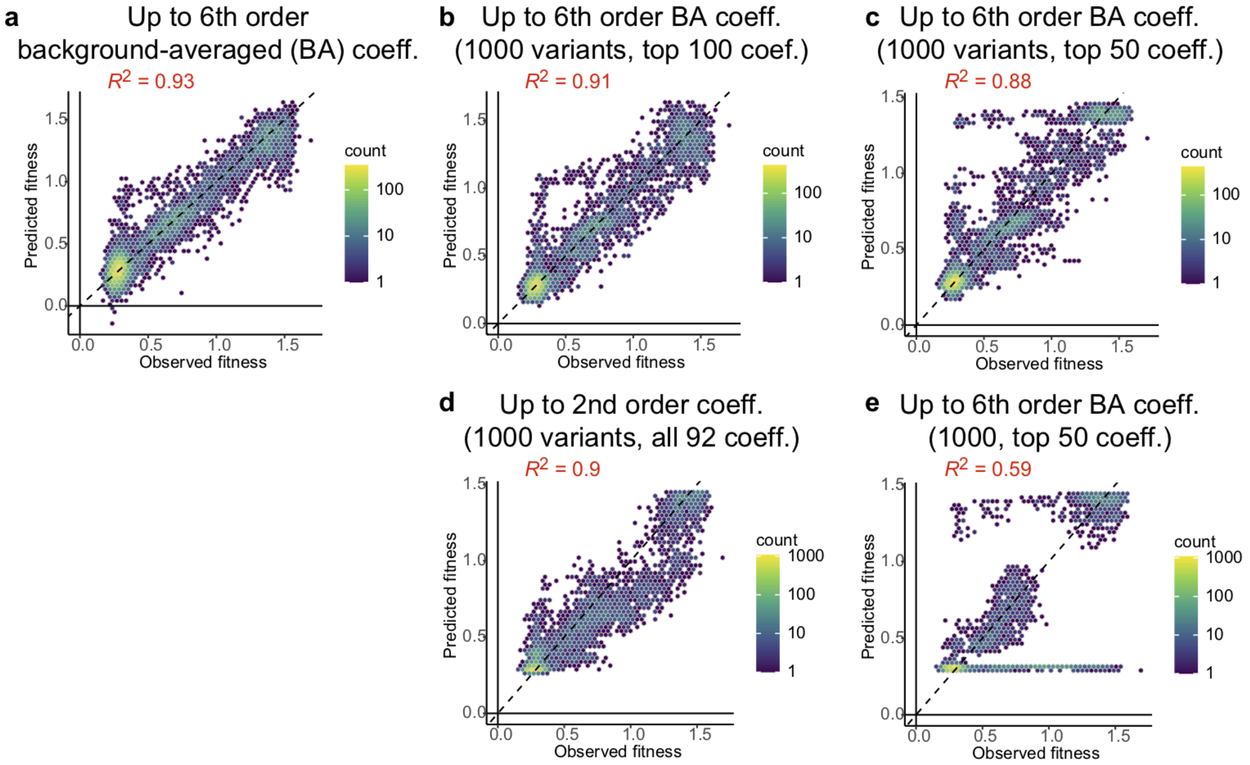

Fig S3. Related to Fig. 5, eqFP611**. a**, Performance of MoCHI model incorporating all background-averaged epistatic coefficients up to 6th order. **b**, Performance of sparse MoCHI model incorporating the top 100 epistatic coefficients including terms up to 6th order trained on a random sample of 1000 variants. **c**, Similar to panel b but model incorporates the top 50 epistatic coefficients. **d**, Performance of MoCHI model incorporating global epistasis and all 92 1st and 2nd order epistatic coefficients. **e**, Similar to panel d but model incorporated the top 50 epistatic coefficients.
